# P*R*O*P: a web application to perform phylogenetic analysis considering the effect of gaps

**DOI:** 10.1186/s12859-021-03978-z

**Published:** 2021-01-30

**Authors:** Takuma Nishimaki, Keiko Sato

**Affiliations:** Department of Information Sciences, Tokyo University of Science, Noda City, Chiba 278-8510 Japan

**Keywords:** Gap information, Genetic difference, Phylogenetic analysis, Web application

## Abstract

**Background:**

Phylogenetic analysis strongly depends on evolutionary models. Most evolutionary models for estimating genetic differences and phylogenetic relationships do not treat gap sites in the alignment of sequences. Appropriately incorporating evolutionary information of sites containing insertions and deletions into genetic difference measures will be improve the accuracy of phylogenetic estimates.

**Results:**

We introduced a new measure for estimating genetic differences, and presented P*R*O*P, a web application for performing phylogenetic analysis based on genetic difference considering the effect of gaps. As an example of phylogenetic analysis using P*R*O*P, we used complete p53 amino acid sequences of 31 organisms and illustrated that the genetic differences with and without information on sites containing gaps result in trees with different topologies.

**Conclusions:**

P*R*O*P is available at https://www.rs.tus.ac.jp/bioinformatics/prop and the user can perform phylogenetic analysis by uploading sequence data on the website. The most distinctive feature of P*R*O*P is its genetic difference that is estimated without eliminating gap sites for alignment sequences, which helps users detect meaningful difference in an evolutionary process. The source code is available in GitHub: https://github.com/TUS-Satolab/PROP.

## Background

Phylogenetic inference based on genetic difference relies on evolutionary models. Most methods for phylogenetic analysis in use today do not treat gap sites in the alignment of sequences. The major reason is probably that evolutionary models with insertions and deletions are more complicated and more challenging than models with only substitutions. However, nucleotide or amino acid changes that have occurred during evolution include substitutions, insertions, and deletions. Therefore, ignoring evolutionary information by insertions and deletions is not appropriate for phylogenetic inference. In a previous paper [1], we have proposed an extension to the Kimura two parameter (K2P) model [2], called K2P + Gap, to incorporate the evolutionary information of sites containing insertions and deletions into the measure for estimating genetic difference between two nucleotide sequences.

Here, we incorporate this idea into the Jukes–Cantor (JC) method [3] for amino acid sequences, and present P*R*O*P (Phylogenetic Relationships based On Proper genetic differences), a web application for performing phylogenetic analysis based on genetic difference considering the effect of gaps in both nucleotide sequences and amino acid sequences. Unlike the software packages for phylogenetic analysis such as MEGA [4] and PAUP* [5], P*R*O*P is a web application, so the user can perform phylogenetic analysis only by preparing sequence data in FASTA format without downloading or installing it.

## Implementation

### Application

The application consists of three steps: execution of sequence alignment, estimation of genetic differences, and generation of phylogenetic tree, which can collectively perform all the steps. On the main interface of P*R*O*P, the user uploads an input sequence file in FASTA format, and, if necessary, specifies the type of sequence, alignment tool, genetic difference measure, treatment of gaps, and phylogenetic tree method. After the processing of the request, pressing the View button allows the user to visualize the generated phylogenetic tree within a frame of its web page. Upon pressing the Download button, the alignment file in FASTA format (alignment.txt), the difference matrix file in PHYLIP format (matrix.txt), and the tree files in Newick format (tree.txt) and Potable Network Graphic format (figtree.png) will be downloaded. The results of phylogenetic analysis are stored in the Asia Pacific (Tokyo) region of Amazon Web Services for seven days.

The length and number of sequences that can be handled in phylogenetic analysis are 3000 or less. The sequences can be aligned with ClustalW2 (Version 2.1) [6] or MAFFT (Version 7.429) [7]. The maximum number of sequences is 1000 when using ClustalW2. For nucleotide sequences, the genetic differences can be calculated using either the P-distance or the K2P measure. For amino acid sequences, the genetic differences can be calculated using either the P-distance or the JC measure. In any case, the user can specify the treatment of gaps (+ Gap/Pairwise Deletion/Complete Deletion). When estimating genetic differences incorporated gap information in P*R*O*P, it is desirable to use the complete gene or protein sequences. Note that otherwise gaps at 5′ or 3′ ends in an alignment may not actually be due to insertions or deletions. The phylogenetic tree can be constructed by either the neighbor-joining (NJ) method [8] or the unweighted pair-group method with arithmetic mean (UPGMA) [9]. The constructed phylogenetic tree is drawn with figtree.js (https://github.com/rambaut/figtree.js). The JavaScript library figtree.js, incorporated into P*R*O*P, provides interactive visualization of phylogenetic trees. Having said that, because its function is limited, tree file in Newick format generated through P*R*O*P can also be visualized and edited with existing software tools.

### An extension of the JC model

The JC measure for estimating genetic difference between two nucleotide sequences, in terms of the number of nucleotide substitutions per site, is estimated by

1 $$K_{{{\text{JC}}}} = - a \,{\text{log}}\left( {1 - \frac{P}{a}} \right),$$

where $P$ is the probability of homologous sites that are different between the two sequences and $a = 3/4$. In the case of $a = 19/20$, Eq. (1) can be used for amino acid sequences [10].

We extend the JC model to estimate genetic differences considering gap information for aligned amino acid sequences. The idea is the same as that for the K2P + Gap difference measure introduced in a previous paper [1]. All amino acid substitutions occur at the same rate $\alpha$ per site per unit time (year). In addition, when each of the twenty amino acids has an equal rate of changing to a gap, the rate of deletions per site per unit time is $\varepsilon$. On the other hand, assuming that a gap changes to one of the twenty amino acids with an equal rate and its rate per site per unit time is $\varepsilon /20$, the rate of insertions (i.e., change of a gap to any of the twenty amino acids) per site per unit time is $\varepsilon$. Therefore, the total rate of amino acid changes per site per unit time $k$ is given by the following mixture:

2 $$k = w\left( {19\alpha + \varepsilon } \right) + \left( {1 - w} \right)\varepsilon { },$$

where $w$ is the mixture weight, which means the probability of amino acid occurrence between two aligned homologous sequences. In such a case, our measure (JC + Gap) for estimating genetic difference between two amino acid sequences, in terms of the number of amino acid changes per site that occurred during $t$ years, is given by

3 $$K_{{{\text{JC}} + {\text{Gap}}}} = 2tk = - \frac{19}{{20}}w\log \left( {\frac{S - P/19}{w}} \right).$$

As described above, in this equation, $w$ is the occurrence probability of amino acids in two sequences compared. $P$ and $S$ are the probabilities of homologous sites showing different amino acids and showing identical amino acids, respectively. Obviously, if gaps do not exist in two sequences compared (namely $w = 1:P + S = 1$), then Eq. (3) becomes equal to Eq. (1).

### Simulation analysis

In order to evaluate the performance of the difference measure in our model (JC + Gap), we investigated the accuracy of phylogenetic reconstruction for both the JC + Gap difference measure and the JC difference measure by using computer simulation. We had 60 model conditions (five numbers of taxa, four sequence lengths, and three change rates) in a similar way to a previous paper [1]. The probability of amino acid substitutions was fixed at 0.01 per site per branch, and the probabilities of insertion and deletion changes were changed to 0.001, 0.002 and 0.005 per site per branch. 100 replications were performed for each model condition. The sequence data corresponding to the leaf nodes on each perfect binary tree were given as input to the phylogenetic reconstruction. For each data set, the JC genetic differences with complete deletion of gaps, the JC genetic differences with pairwise deletion of gaps, and the JC + Gap genetic differences were estimated to reconstruct phylogenetic trees using the NJ method (see [1] for more details).

## Results

### Accuracy of phylogenetic reconstruction

**Fig. 1:**
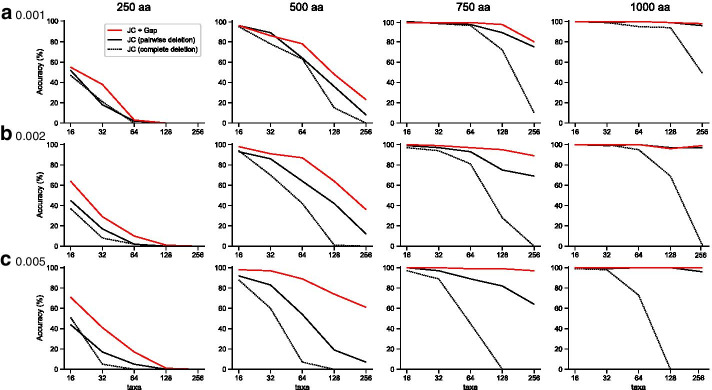
Comparison of accuracy of phylogenetic reconstruction. For model trees of 16, 32, 64, 128, and 256 taxa, ancestral sequences of 250, 500, 750, and 1000 amino acids in length were randomly generated under the following conditions: the probability of amino acid substitutions was fixed at 0.01 per site per branch, and the probabilities of insertion and deletion changes were changed to **a** 0.001, **b** 0.002 and **c** 0.005 per site per branch. The accuracy of phylogenetic reconstruction was evaluated as the percentage of replications in which the correct topology was obtained when compared to the model tree

The average percentage of correctly reconstructed topologies in data sets for all 60 model conditions was 46.1% when calculated with the JC difference measure (complete deletion), 64.2% when calculated with the JC difference measure (pairwise deletion) and 73.3% when calculated with the JC + Gap difference measure (Fig. 1). In case the probabilities of insertion and deletion changes were 0.001, the average accuracy for the JC difference measure (complete deletion), the JC difference measure (pairwise deletion) and the JC + Gap difference measure was 56.6%, 65.9% and 69.9%, respectively. In case of 0.002, the corresponding average accuracy was 46.0%, 64.4% and 72.8%, respectively. In case of 0.005, the corresponding average accuracy was 35.6%, 62.4% and 77.2%, respectively. Moreover, the average percentage of correctly reconstructed topologies in data sets for all 45 model conditions except for sequences of 250 amino acids in length, was 57.6% when calculated with the JC difference measure (complete deletion), 81.1% when calculated with the JC difference measure (pairwise deletion) and 90.4% when calculated with the JC + Gap difference measure. The JC + Gap difference measure shows the highest accuracy of the three measures.

### Phylogenetic analysis

**Fig. 2:**
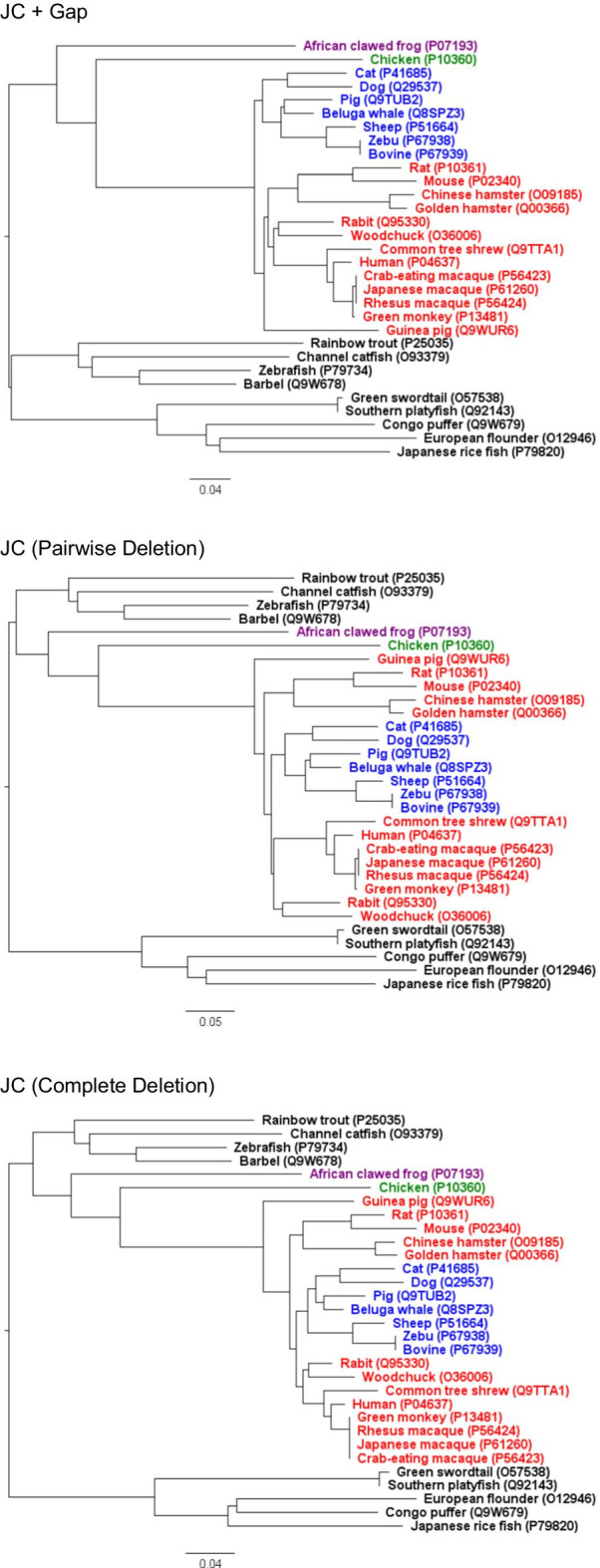
Phylogenetic trees of the p53 amino acid sequences based on the treatment of gaps “+Gap”, “Pairwise Deletion” and “Complete Deletion”. Each tree was generated using P*R*O*P and was midpoint rooted using FigTree. Organism species are colored as follows: Actinopterygii (9 species), black; Amphibia (1 species), purple; Aves (1 species), green; Mammalia Euarchontoglires (13 species), red; Mammalia Laurasiatheria (7 species), blue

We introduced a new measure for estimating genetic differences, and presented P*R*O*P, a web application for performing phylogenetic analysis based on genetic difference considering the effect of gaps. Here, we use the amino acid sequences of cellular tumor antigen p53 as an example to illustrate the effect of different treatment of gaps in phylogenetic analysis using P*R*O*P. Complete p53 amino acid sequences of 31 organisms were retrieved from UniProt KB/Swiss-Prot database (https://www.uniprot.org/uniprot/). These 31 sequences with amino acid length ranging from 352 to 396 were aligned with MAFFT and the genetic differences were respectively calculated using the JC measure in each case of the treatment of gaps (+Gap/Pairwise Deletion/Complete Deletion). For each of the three cases, the phylogenetic tree was generated with the NJ method and the resulting Newick tree file was furthermore plotted and edited in FigTree (Version 1.4.4) developed by Andrew Rambaut. The p53 sequences were grouped according to their class (Actinopterygii, Amphibia, Aves, and Mammalia) in all three trees; however, as for the class Mammalia, the tree based on the JC + Gap difference measure had a different topology compared to the other two trees (Fig. 2). Two subtrees in its tree based on the JC + Gap difference measure that are rooted at the sibling nodes of the same internal node correspond to the two clades (Euarchontoglires and Laurasiatheria), respectively. Many studies support that the superorder Euarchontoglires and the superorder Laurasiatheria are sister taxa [11–15]. The result with the JC + Gap difference measure in our analysis is consistent with these studies.

## Conclusions

P*R*O*P is a web application for performing phylogenetic analysis based on genetic difference considering the effect of gaps. The user can perform phylogenetic analysis by uploading sequence data in FASTA format. The most distinctive feature of P*R*O*P is its genetic difference that is estimated without eliminating gap sites for alignment sequences, which helps users detect meaningful difference in an evolutionary process and obtain a more accurate classification. The front-end is implemented in JavaScript using the Angular framework. The back-end is implemented in Python and is deployed on the Amazon Elastic Compute Cloud (Amazon EC2). P*R*O*P is available at https://www.rs.tus.ac.jp/bioinformatics/prop. We will continue to update P*R*O*P by adding additional information, improving the implementation, and incorporating new measures for estimating genetic differences. The user can always access the latest version of P*R*O*P.

## Availability and requirements

- Project name: P*R*O*P.
- Project home page: https://www.rs.tus.ac.jp/bioinformatics/prop.
- Operating system(s): Platform independent (web-based).
- Programming language: JavaScript and Python.
- Other requirements: Not specified.
- License: MIT license.
- Any restrictions to use by non-academics: None.

## Data Availability

The P*R*O*P web interface is freely available at https://www.rs.tus.ac.jp/bioinformatics/prop. The source code can be found at https://github.com/TUS-Satolab/PROP.

## Abbreviations

K2P: Kimura two parameter
JC: Jukes–Cantor
NJ: Neighbor-joining
UPGMA: Unweighted pair-group method with arithmetic mean
